# Sung Shu Chien: the founder of modern Chinese botany

**DOI:** 10.1093/procel/pwac047

**Published:** 2022-10-27

**Authors:** Huan Liu, Kaijing Huang, Xuefan Yuan, Hao Cheng

**Affiliations:** University of Science and Technology of China, Hefei 230026, China; State Key Laboratory of Virology, Wuhan 430072, China; University of Science and Technology of China, Hefei 230026, China; Nanjing University of the Arts, Nanjing 210013, China; Institute of Microbiology, Chinese Academy of Sciences, Beijing 100101, China

Sung Shu Chien (钱崇澍, 1883–1965) (Fig. 1) was a famous botanist and educator in China, one of the founders of Chinese plant taxonomy, plant physiology, geobotany, and floristics. He devoted his whole life to scientific research and education, and was a founder of modern Chinese botany. Sung Shu Chien pioneered several research fields in modern Chinese botany. In 1916, he published the first literature in Latin on the naming and classification of plants. In the following year, he published the earliest thesis on the application of modern scientific methods of plant physiology in China. He wrote the first paper in the area of plant ecology and geobotany in China. He was a pioneer in the systematic study of the families Orchidaceae, Urticaceae, Fabaceae, and Ranunculaceae in China. From 1959 to 1965, he was the chief editor of “Flora of China” and was responsible for the section on Urticaceae (Yu, 1983). Sung Shu Chien initiated the research of plant taxonomy, plant physiology, and plant ecology in China, and laid a solid foundation for the extensive research of vegetation, plant zoning, and plant specialties in China (Chien et al., 1956). By skillfully combining research and education, he cultivated Chinese scientific and technological talents in botany.

**Figure 1.**
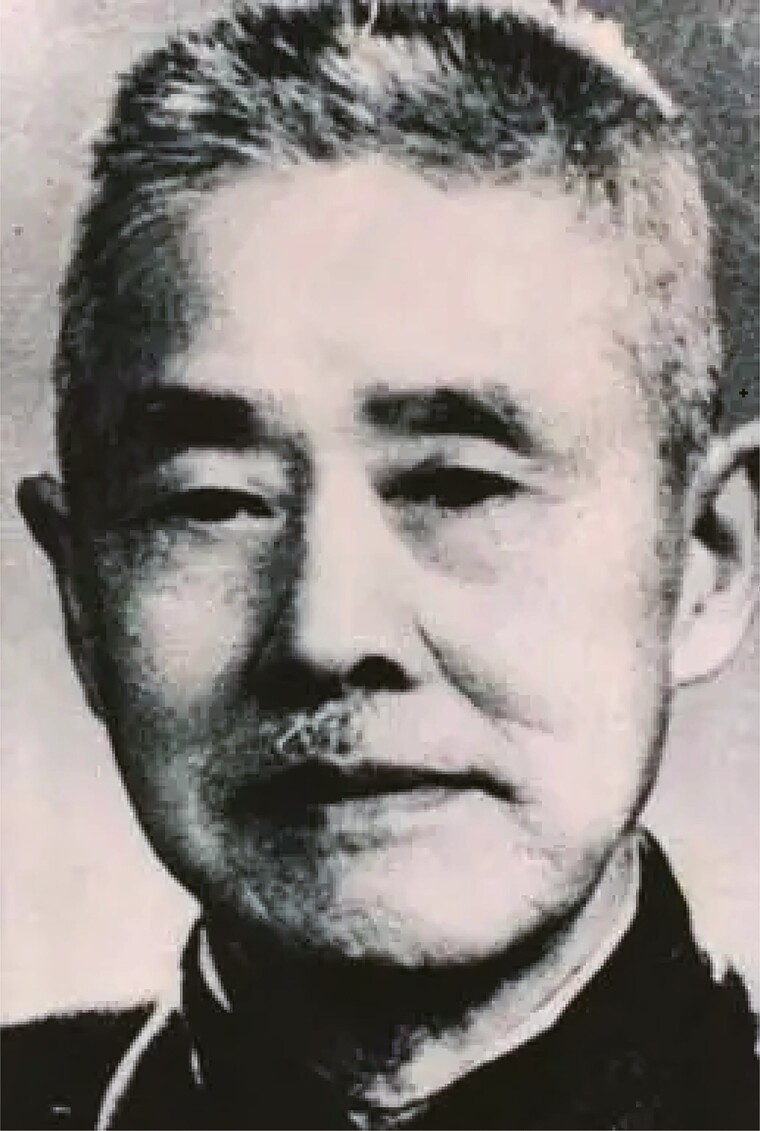
Sung Shu Chien—the founder of modern Chinese botany.

## Studying and working life

Born in Zhejiang Province, Sung Shu Chien was diligent since childhood under the cultivation of a well-educated family. He became a scholar in the last imperial examination held in the Qing Dynasty in 1904. Then, he studied new sciences in response to the demands of the times. In 1905, he was admitted to Shanghai Nanyang Public School (the predecessor of Xi’an Jiaotong University and Shanghai Jiao Tong University). In 1910, he was admitted to the Tsinghua University Preparatory School (the predecessor of Tsinghua University) as a state-funded student. He then went to the USA for further study together with 70 other students, including Hu Shih, Siguang Li, and Kezhen Zhu. He first studied agronomy at the College of Science of the University of Illinois. After 1 year, he transferred to the College of Natural Sciences of the University of Illinois, majoring in botany, and graduated with a Bachelor of Science degree in July 1914. Because of his strong interest in botany, he chose to continue his studies at The University of Chicago and Harvard University, where he studied plant physiology, plant ecology, and plant taxonomy, and received his master’s degree from The University of Chicago (Wang, 1984).

**Figure 2.**
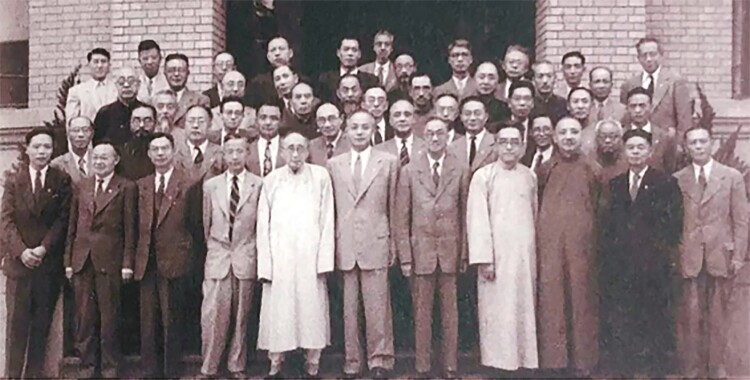
Sung Shu Chien (sixth from left in the back row) at the first Convocation Meeting of Academia Sinica in 1948.

In 1916, Sung Shu Chien returned to China and worked as a professor in multiple universities including Beijing Agricultural University (the predecessor of China Agricultural University), Tsinghua University, and Fudan University. In 1923, he collaborated with Bingwen Zou and Hu Hsien-Chien to write China’s first biology textbook, “Advanced Botany.” In 1926, he served as the first dean of the Department of Biology of Tsinghua University. In 1948, Sung Shu Chien was elected as an Academician of Academia Sinica (Fig. 2). In 1955, Chien was elected as one of the first members of the Chinese Academy of Sciences (Liu, 1981).

## Founder of botany in China

China is a vast country with rich vegetation and a wide variety of rare species. However, in the 19th century, Chinese botanical research was dominated by foreigners for a long time, a large number of rare and model specimens were shipped abroad, botanical literature was rare, and local agriculture was lagging behind the world. Reacting to this situation, Sung Shu Chien was determined to establish modern botany in China and improve the agricultural development of the country.

**Figure 3.**
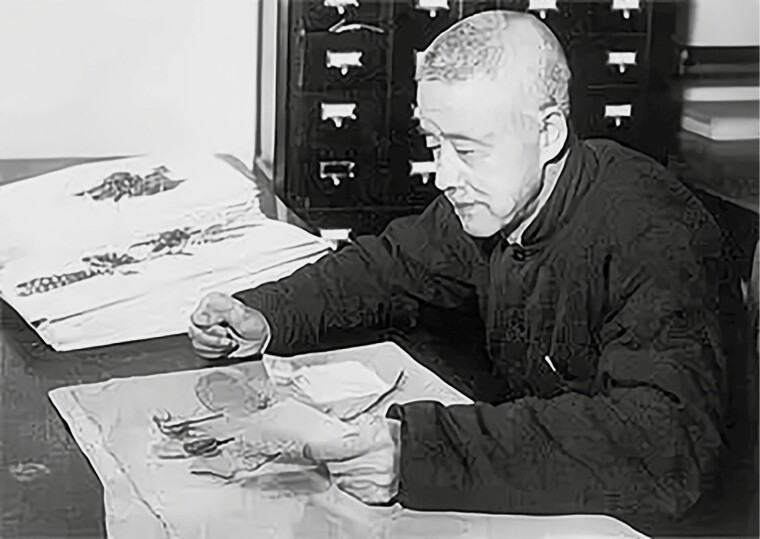
Sung Shu Chien at work.

Sung Shu Chien initiated research in the fields of plant taxonomy, plant physiology, and plant ecology in China. His early works and translations filled the previously blank pages of botany in China. Many of his papers were praised by domestic and foreign academic communities. In 1916, Sung Shu Chien published “Two Asiatic Allies of *Ranunculus pensylvanicus,*” which is the first paper written by a Chinese author using Latin language for the plant name and classification. This paper marked the birth of modern plant taxonomy in China (Liu, 1981). During his taxonomic studies at Harvard University, he also showed a keen interest in plant physiology. He conducted experiments demonstrating the effects and mechanism of salt absorption by cells proposed at that time under the advice and guidance of Professor W. J. V. Osterhout (Osterhout, 1935). He published the first independent application of modern scientific methods to study plant physiology by a Chinese author in the Botanical Gazette in 1917 (Wang, 1984). His paper titled “Peculiar Effects of Barium, Strontium, and Cerium on Spirogyra,” initiated plant physiology in China (Chien, 1917). In 1927, he completed the first paper on Chinese geobotany and zonal botany, “A Preliminary Study of the Vegetation System of Anhui Huangshan.” In 1929, he translated literature on plant physiology such as “Osmotic Properties of Cells” and “Photosynthesis in Autotrophic Plants” (Liu, 1981) (Fig. 3).

## A new genus of orchids from China

Sung Shu Chien contributed to the taxonomy of Orchidaceae, Urticaceae, Fabaceae, and Ranunculaceae. Orchidaceae is the largest family of Monocotyledons. At present, there are >800 genera and nearly 25,000 species of orchids in the world, including ~194 genera and 1388 species in China. Under natural conditions, orchid plants are prone to hybridization, which has led to the occurrence of many interspecific and even intergeneric hybrids. At the same time, orchids have great variability, leading to the creation of many intermediate types and poorly defined species boundaries (Yang et al., 2005). Urticaceae, Fabaceae, and Ranunculaceae also have multiple unclearly separated genera and interspecific hybridization, causing great difficulties for the classification and systematic study of these plants. In the early 20th century, when China lacked relevant specimens and literature, Sung Shu Chien resolutely chose to devote himself to the systematic study and classification of these important plant families in China.

**Figure 4.**
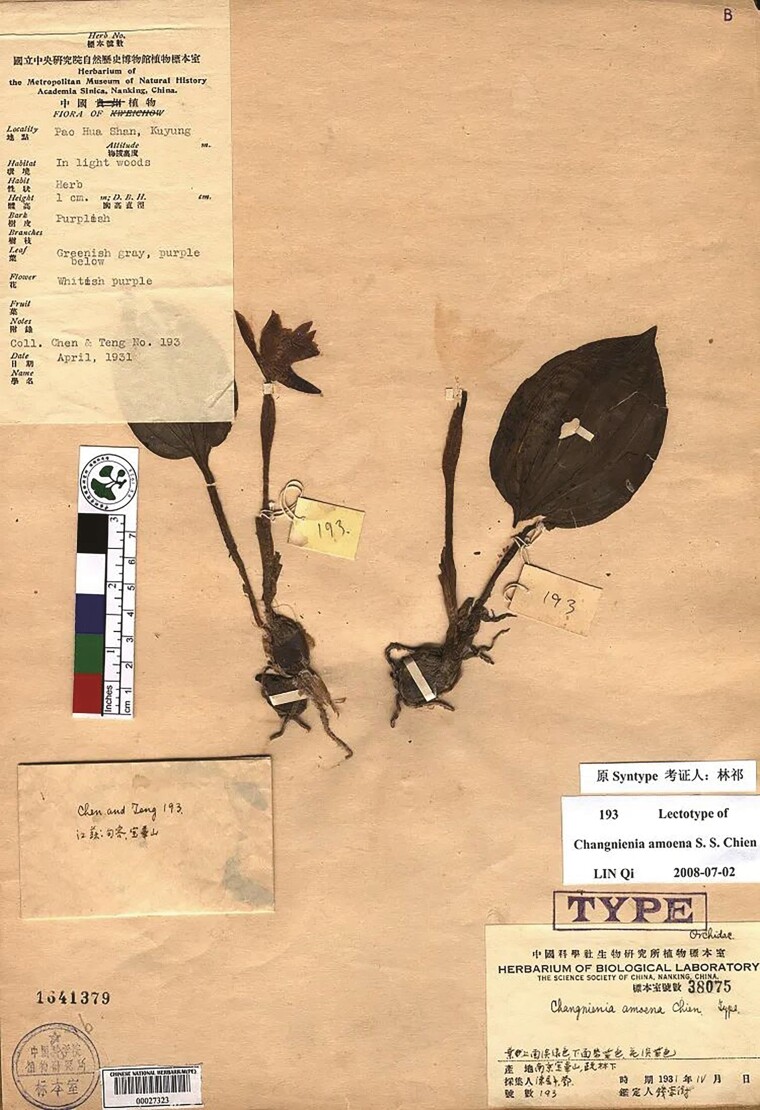
Type specimens of *Changnienia amoena* S.S. Chien.

*Changnienia amoena* S.S. Chien is a national second-class protected plant from the family Orchidaceae, and a rare species in China. It was first discovered and collected by Changnian Chen and Shiwei Deng in 1931 on Baohua Mountain, Guangxi, China (Fig. 4). In 1935, Sung Shu Chien, who was then a professor and director of the Botany Department at the Biological Laboratory of the Science Society of China, noticed and verified that this was a new species and even a new genus of orchids. The corresponding paper titled “A New Genus of Orchids from Eastern China” was published in the Contributions from the Biological Laboratory of Science Society of China, Botanical Series, announcing the discovery of a new genus and species, which was named *C. amoena* S.S. Chien. The paper documented the unique biological characteristics of *C. amoena* S.S. Chien and pointed out that the new genus can be distinguished from the similar *Calypso* species based on the leaf characteristics, especially the labellum: the former has a distinctive spine-like shape on the labellum, while the latter does not (Chien, 1935).

## “Flora of China”

In the 1960s, Sung Shu Chien devoted himself to the enormous and arduous but historically significant and valuable task of compiling the “Flora of China.” This publication contains important scientific information such as nominal classification, morphological characteristics, and systematic locations in plant taxonomy. The compilation of this book required vast knowledge of Chinese plant specimens overseas, as well as detailed observation and study of domestic plant specimens, which were difficult at the time. The type specimens of many native species were overseas even though China has high plant species richness.

**Figure 5.**
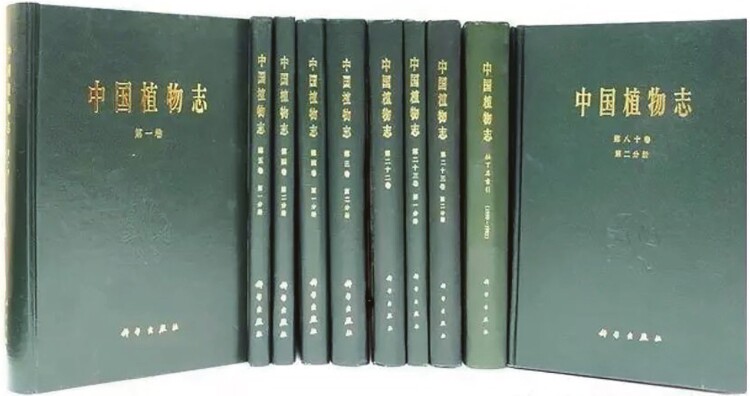
“Flora of China”.

During his studies at Harvard University, Sung Shu Chien often visited the Gray Herbarium and the Herbarium of Arnold Arboretum, which had a large collection of Chinese plant specimens. He closely observed the Chinese native specimens, acquiring knowledge of plant taxonomy and information on indigenous plants (Wang, 1984). After returning to China, he conducted a thorough field investigation of Zhejiang and southern Jiangsu, in which he collected >10,000 plant specimens for analysis and research, and then investigated and collected many plant specimens from Sichuan, Anhui, and other places, preparing for the Flora of China. In October 1959, after >40 years of accumulation, the editorial committee of “Flora of China,” led by Sung Shu Chien, was formally established, setting up a great project in the science and technology history of botany. Sung Shu Chien led the compilation of “Flora of China” until 1965, and took charge of the edition on the Urticaceae. During his tenure as chief editor, three volumes of “Flora of China” were published (Liu, 1980). In 2004, the world’s largest flora, which has been compiled by four generations of Chinese botanists over a period of 80 years, was finally completed, demonstrating to the world the great progress of modern botany in China (Fig. 5). In 2009, the “Flora of China” received the first prize of The State Natural Science Award (Hong et al., 2008).

## The first botany textbook and the first nature reserve

Sung Shu Chien realized that basic research is essential to establish botany in China. In 1922, he worked with Hu Hsien-Chien to establish a botany department at the Biological Laboratory of the Science Society of China, setting up a laboratory, herbarium, and library that met scientific standards, which facilitated the development and promoted the research work of native botany. In 1937, Chien et al. in the Biological Laboratory of the Science Society of China moved to Beibei, Chongqing, and continued scientific work even in the hardest war time (Yu, 1983).

**Figure 6.**
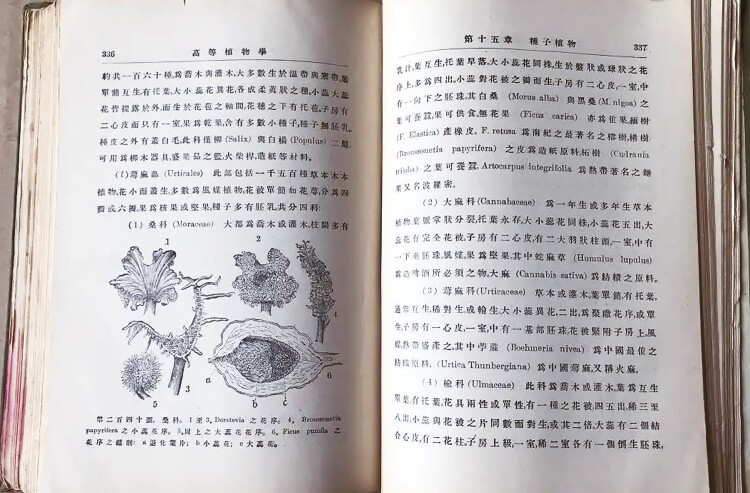
The first edition of “Advanced Botany” in 1923.

Sung Shu Chien always paid attention to both scientific research and education. In 1923, he co-edited the book “Advanced Botany” with Zou Bingwen and Hu Hsien-Chien (Fig. 6). It was the first botany textbook in China, which corrected the mistakes of previous books and established a complete knowledge system, filling the gaps of Chinese botany textbooks and significantly promoting botanical education in China (Yu, 1983).

Through intensive study of plant ecology and fieldwork, Sung Shu Chien realized the preciousness of China’s natural resources and the threat they faced. Perspectively, he proposed to strengthen the protection and rational use of natural resources such as forests. In 1956, he jointly submitted a proposal to the National People’s Congress of the People’s Republic of China on the initiatives of non-logged areas of the country, which led to the establishment of China’s first Nature Reserve on Dinghu Mountain, Guangdong Province. Since then, the establishment of Nature Reserves in China was initiated (Zhu et al., 1993).

## “A Resolve Makes A Will”

In a notable article, Sung Shu Chien stated that “A Resolve Makes A Will”: when China was suffering, “I was influenced by the idea of serving the country through science, and was not interested in pursuing authority or fortune, but was determined to devote myself to science”; after the People’s Republic of China was founded, “I was so inspired and worked hard for the development of China’s scientific cause.” His true talent and modesty have won him high praise and admiration. “He was a scientist and educator with high moral character, love for the motherland, willingness to help others, qualities of rigorous study, and courageous dedication,” said Dejun Yu, a plant taxonomist and vice chairman of the Institute of Botany of the Chinese Academy of Sciences, in his speech on the 100th anniversary of Sung Shu Chien’s birth (Yu, 1983). As a pioneer and outstanding educator, Sung Shu Chien’s academic achievements have set milestones of modern Chinese botany. His pioneering approach to plant classification and research will continue to inspire future generations to advance botany, and his dedication to the development of modern science in China will be forever remembered.

## References

[CIT0001] Chien SS. Peculiar effects of barium, strontium, and cerium on spirogyra. Bot Gaz 1917;63:406–409.

[CIT0002] Chien SS. A new genus of orchids from Eastern China. Contr Biol Lab Sci Soc China Bot Ser 1935;10:89–92.

[CIT0003] Chien SS , Wu ZY, Chen CD. Vegetation types in China. Acta Geogr Sin 1956;22:37–92. [钱崇澍, 吴征镒, 陈昌笃 (1956) 中国植被的类型. 地理学报 22: 37–92]

[CIT0004] Hong DY , Chen ZD, Qiu YL. History inspires progress, future inspires hope: commemorating the 75th anniversary of the Chinese Botanical Society. J Syst Evol 2008;46:439–440. [洪德元, 陈之端, 仇寅龙 (2008) 历史催人奋进, 未来令人憧憬—纪念中国植物学会成立75周年. 植物分类学报 46 (4): 439–440]

[CIT0005] Liu CZ. The famous botanist: Sung Shu Chien. Plants 1980;(5):18–20. [刘昌芝 (1980) 著名植物学家钱崇澍先生. 植物杂志,(5):18–20]

[CIT0006] Liu CZ. The pioneer of modern botany—Sung Shu Chien. Chinese J Hist Sci Technol 1981;(3): 35–39. [刘昌芝 (1981) 近代植物学的开拓者——钱崇澍. 中国科技史料,1981;(3):35–39]

[CIT0007] Osterhout WJV. Mechanism of salt absorption by plant cells. Nature 1935;136:1034–1035.

[CIT0008] Wang ZR. The initiator of plant physiology in China - Sung Shu Chien. Plant Physiol J 1984;2:62–64. [汪振儒 (1984) 我国植物生理学的启业人钱崇澍先生. 植物生理学通讯2:62–64]

[CIT0009] Yang ZJ , Zhu GF, Zhang X. Advances in the systematics and affinities of orchids. Acta Bot Boreal-Occid Sin 2005;09:194–199. [杨志娟, 朱根发, 张显 (2005) 兰科植物系统学及亲缘关系研究进展. 西北植物学报09:194–199]

[CIT0010] Yu DJ. Commemoration of the 100th anniversary of the birth of Professor Sung Shu Chien, the founder of modern botany in China. J Integr Plant Biol 1983;25:495–496. [俞德浚 (1983) 纪念我国近代植物学的奠基人—钱崇澍教授诞辰一百周年. 植物学报 25 (5):495–496]

[CIT0011] Zhu KZ , Chien SS, Bing Z et al. Views on the destruction of natural resources and suggestions on the future enhancement of rational utilization and protection. China Popul Resour Environ 1993;1:77–81. [竺可桢, 钱崇澍, 秉志等 (1993) 关于自然资源破坏情况及今后加强合理利用与保护的意见. 中国人口∙资源与环境1:77–81]

